# Social Media Discussions of Anti-Diabetic Drugs and the Popularity of GLP-1 Therapies: Content Analysis

**DOI:** 10.2196/87590

**Published:** 2026-08-26

**Authors:** Chloe Stallion, Tamkeen Khan, Christian Guerrero, Stavros Tsipas, Gregory Wozniak

**Affiliations:** 1 American Medical Association, 330 N. Wabash Ave, Suite 39300, Chicago, IL, 60611, United States, 1 312-464-5227

**Keywords:** diabetes, obesity, insulin, Twitter, social media, infodemiology, infoveillance, social listening, cost, rationing, weight loss, GLP

## Abstract

**Background:**

The clinical use of antidiabetic drugs (ADDs) has gained prominent public visibility due to the Food and Drug Administration (FDA) expansion of glucagon-like peptide-1 receptor agonists (GLP-1 RAs) in recent years; these drugs are now widely prescribed for weight loss. This shift is reflected in online public discourse.

**Objective:**

This study aimed to analyze the current online public discourse surrounding ADDs, with an emphasis on perceptions of insulin versus noninsulin therapies and the increasing prominence of weight loss–associated medications.

**Methods:**

We conducted a retrospective analysis of 23,580 English-language posts from the United States in 2023, using Boolean keyword searches to examine conversations about insulin-inclusive, with or without weight loss terms, and insulin-exclusive, with or without weight loss terms. We analyzed the volume and sentiment of online conversations regarding ADDs, frequency of drug mentions, proportion of posts by self-identified physicians, and thematic analysis of patient and clinician concerns.

**Results:**

A total of 26,832 initial posts were collected in 2023. After post validation, 23,580 posts remained as the study sample size. Of the 23,580 posts, 4.6% (1087/23,580) mentioned insulin, and 95.4% (n=22,493) did not mention insulin. Semaglutide-containing drugs such as Ozempic and Mounjaro were the most referenced medications, particularly in weight-loss contexts. Weight loss conversations made up the majority of online posts. Conversations about insulin were marginal compared with conversations that did not mention insulin online. Only 7% to 10% of posts came from self-identified physicians. Key themes included drug accessibility, off-label use for weight management, concerns about supply shortages, insurance coverage, and growing calls for holistic care. Notably, public perspectives favored the dual efficacy of medications like Ozempic in managing diabetes and promoting weight loss.

**Conclusions:**

The discourse on ADDs is dominated by weight loss–oriented therapies, with GLP-1 RAs driving much of the engagement. This surveillance paper illustrates the current state of public health interests around antidiabetic medications associated with weight loss and raises concerns regarding equitable access for patients with diabetes. These findings underscore the need for updated clinical guidance on necessary lifestyle behaviors for antidiabetic medication use and ongoing monitoring of public opinions regarding chronic disease management medications.

## Introduction

Diabetes affects 38.1 million adults (14.7%) in the United States [1]. Diabetes is a significant risk factor for heart disease, stroke, and kidney problems [2].

Antidiabetic drugs (ADDs) are vital for managing type 2 diabetes by maintaining blood glucose levels [3]. Traditionally, insulin-based therapies were the mainstay; however, recent years have seen a surge in noninsulin options, such as glucagon-like peptide-1 receptor agonists (GLP-1 RAs) [1,3,4].

The weight loss side effect associated with taking these drugs has made certain medications a consideration for patients who struggle to lose weight but do not have type 2 diabetes [5]. In particular, 2 GLP-1 RA molecules, semaglutide and tirzepatide, are indicated for type 2 diabetes but have also been approved for weight loss, under different brand names [6,7]. Semaglutide was Food and Drug Administration (FDA)–approved to treat type 2 diabetes in 2017 [8]. These medical advancements in weight loss have become major topics within the diabetes conversation online. Physicians also prescribe semaglutide off-label to patients to help promote weight loss, which has increased the demand for diabetes medications [9-11]. With FDA approval of tirzepatide to treat type 2 diabetes in the spring of 2022, there was a 400% increase in ADD prescriptions by nonendocrinologists, and the drug was subsequently approved for weight loss [7,12].

Earlier studies have explored the public perception of ADDs, showing an overall positive opinion driven by diabetes programs and patients [13]. With more recent approvals for specific GLP-1 RAs to be used for weight management, there is an increased demand for these medications for reasons other than diabetes management [12,14]. Total spending on GLP-1 RAs increased by more than 500% from 2018 to 2023 [15], and state-level spending and fills were higher in states with higher obesity prevalence [16]. This study aimed to analyze the notable online discourse surrounding ADDs, emphasizing the growing preference for weight loss–associated therapies over traditional insulin-based treatments as seen in online conversations. Antidiabetic medications have become a popular topic of interest, and monitoring online conversation shows that these medications are largely discussed in association with weight loss in nondiabetic patients. As a surveillance study, our aim was to monitor and report on the current online discourse surrounding antidiabetic medications.

## Methods

### Overview

This methodology provides insight into the current use of antidiabetic medications available in the marketplace. Although this study did not evaluate causal mechanisms, observations of the most frequently mentioned drugs may be associated with factors such as the availability of pharmacological options, direct-to-consumer marketing, perceived efficacy, and affordability. The methodological approach applied in this surveillance study offers a foundation for future research to systematically assess these potential influences. We created a query to collect all publicly available posts posted between January 1, 2023, and December 31, 2023, on X (formerly Twitter). The methodology involved developing a structured query using a targeted set of keywords and phrases related to the study of ADD use. These terms were entered in various combinations, accounting for variations in spelling, brand and generic drug names, and contextual keywords to improve precision. There were 2 distinct queries: one designed to capture conversation around insulin, and another excluding insulin. This approach enabled a nuanced analysis of perspectives, themes, and engagement patterns across different audience segments.

### Query Structure

The query structure shown in Multimedia Appendix 1 has 2 main queries: query 1 and query 2, which mirror the previous study [13]. Query 1 explores ADDs conversations including insulin, and query 2 explores ADDs conversations excluding insulin. This updated study expands each of those queries by adding a subquery that differentiates conversations including weight loss and conversations excluding weight loss. Query 1a in query 1 of the study explores ADDs and insulin but excludes weight loss–associated ADDs; query 1b in query 1 of the study explores only weight loss–associated ADDs and insulin conversations. In contrast, query 2a in query 2 of the study explores ADDs, excluding insulin, but includes weight loss–associated ADDs; query 2b in query 2 explores only weight loss–associated ADDs and excludes insulin. The general keywords related to diabetes and insulin were kept consistent with the previous study, using Boolean language to include or exclude insulin. The ADD terms from the previous study were separated into those not associated with weight loss and those associated with weight loss and were used for query 1b and query 2b, respectively.

### Keyword Selection

Keywords were selected using the query terms, which included search terms associated with diabetes, insulin, and ADDs (Multimedia Appendix 2). ADDs are identified by their generic and brand names. Certain drugs are sold under one FDA-approved name, and others are sold under multiple FDA-approved brand names; therefore, the search terms included generic names, brand names, and common misspellings. (Multimedia Appendix 3). Expanding upon the previous study, this study aimed to explore differences in online conversations between drugs not associated and those positively associated with weight loss. Additional terms added in this study include *BMI and A_1c_* to further separate the data into those concerning and not concerning weight loss; however, because health care professionals largely use these terms, they excessively limited the scope of the query when used as exclusionary terms. Instead, they were used as supplementary terms for diabetes.

### Query Filtering

Topic modeling was conducted on each query to identify noise that entered the initial results and did not pertain to the topic outlined in the study. Keywords associated with the noise were excluded from the query results, which yielded a body of posts within each query that focused on antidiabetic medication use without noise.

The posts were filtered to remove results from protected (private) X accounts, in which post content data were not visible. Additional filters removed posts originating outside the United States and those in languages other than English. Original posts were the primary focus for understanding the online conversation; therefore, reposts and quote posts were excluded from the query. To best capture the breadth of the conversation, only original posts were included. Finally, 8 noise categories—advertisements, autoposts, contests and games, financial news, scams, job offers, not safe for work, and vulgar content—were excluded from the query to limit irrelevant conversation.

### Descriptive Data Analysis

#### ADD Counts

Drug counts were created by calculating the number of mentions of each drug across every query. In the online space, both the chemical names and the brand names are used to identify ADDs. The chemical compounds and their brand-name counterparts were combined for each drug count, except for semaglutides, since compounded semaglutide is distinct from brand-name semaglutides. Different drug terms were used within each query at varying rates, highlighting which medications were more widely used and sought after than others. Brand names and generic names for these medications were used interchangeably online; therefore, generic drugs that were sold under a specific FDA-approved brand name were grouped as one term (eg, Victoza and liraglutide). Conversely, generic drug names that are sold under multiple FDA-approved brand names are counted separately (eg, Mounjaro, Zepbound, and tirzepatide).

#### Physician Counts

Posts made by clinicians were identified by “md” and “medical doctor” keywords in the author’s name and username (Multimedia Appendix 4). The term “doctor” in isolation was not used as a query keyword because it encompasses far too large a range of nonmedical professions (eg, PhD). Variations of the abbreviated forms of “medical doctor” were used to account for common spellings. Misspellings of “medical doctor” or “med doc” were not included. These posts were manually filtered to remove false-positive individuals and medical organizations as this study focuses on clinicians (ie, physicians).

### Qualitative Content Analysis

A proprietary natural language processing algorithm created by Ipsos using Google BERT’s AI language model was used to categorize opinions and perspectives [17]. Physician-related posts were identified through keywords indicating medical credentials. These exclusion criteria allowed for a more precise manual evaluation of authentic conversations by ensuring that only organic, user-generated discussions were analyzed. Manual analysis was performed to group themes for each query and subquery within the study.

### Ethical Considerations

Because no individuals were put at risk during this data collection process, this study did not meet the requirements to be considered human subjects research. The data for this project were collected through a web scraping of public X data powered by Ipsos and did not require any interaction with human participants. We observed and aggregated publicly available data. The American Medical Association partnered with the University of Illinois-Chicago for institutional review board processes. Their determination is provided below:

Because you are not engaging with human subjects and all of the data being analyzed is publicly available information, this research would not qualify as human subjects research.

## Results

### Volume of Posts

**Figure 1.**
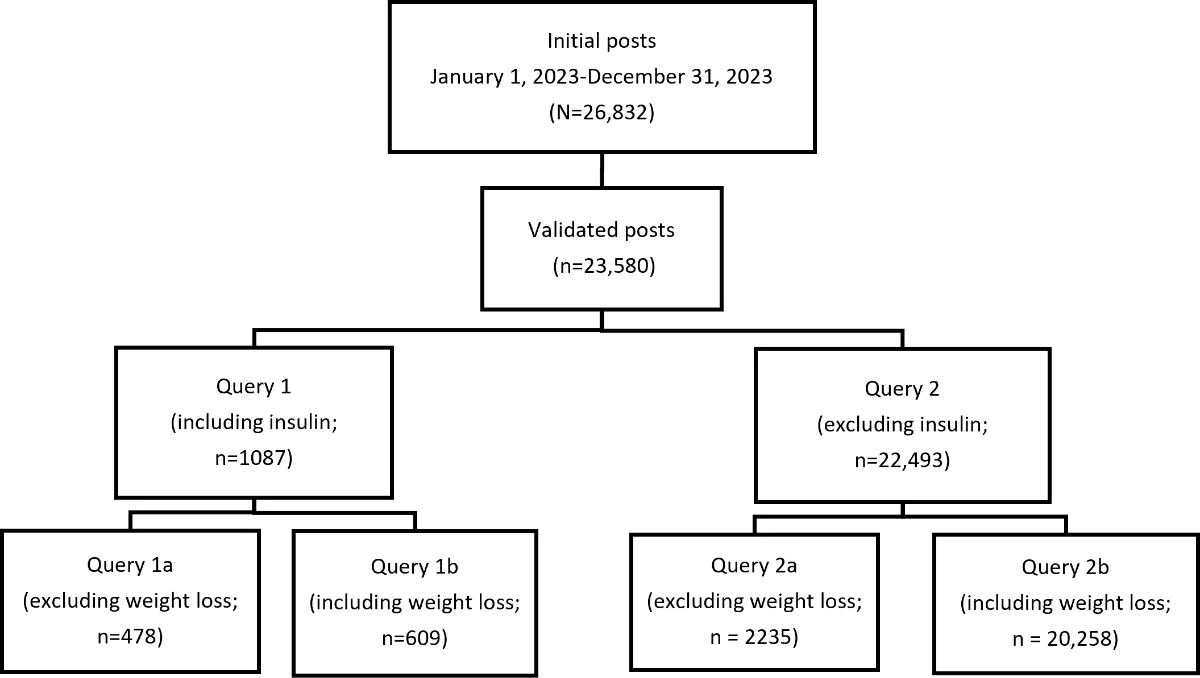
Flowchart of post validation and categorization.

The number of posts for the 4 queries varied over the observation period, as shown in Figure 1, with more insulin-related diabetes medication conversations discussing weight loss than conversations that did not discuss weight loss. Query 2 (excluding insulin conversation) has a much larger number of posts than query 1, encompassing 95.4% (22,493/23,580) of the validated posts, as seen in Figure 1. Within query 2, the overwhelming majority (20,258/22,493, 90%) of the posts discussed weight loss. Noninsulin ADDs associated with weight loss made up most of the diabetic conversations online. This suggests that most of the conversations online surrounding diabetes do not include insulin-related treatments and instead focus on ADDs that are associated with weight loss. These results illustrate that weight loss–associated with ADDs currently dominated online diabetes conversations.

### Drug References

**Figure 2.**
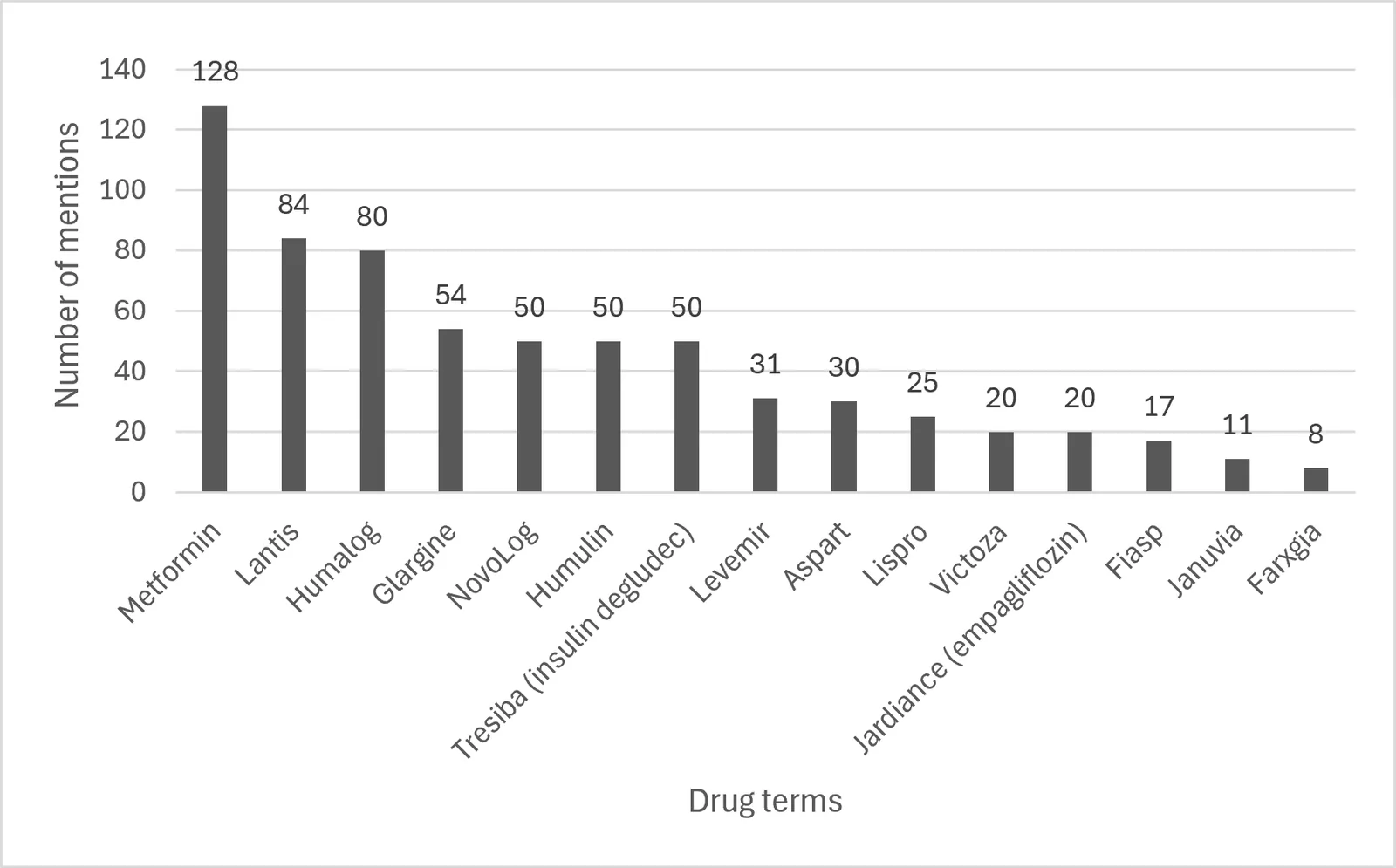
Top 15 drug terms mentioned in query 1a.

Figure 2 shows query 1a (including insulin and excluding weight loss) contained 39 specific ADDs. Of those ADDs, metformin (n=128) had the most references online, followed by Lantus (n=84), a long-acting insulin product, which had the next most references. Humalog (n=80) and Novolog (n=50), both rapid-acting insulin medications, followed in online popularity. All the drugs listed above can be used to treat type 1 and type 2 diabetes, except metformin, which is FDA-approved to treat only type 2 diabetes. Metformin was mentioned in 27% (128/478) of the posts in query 1a.

**Figure 3.**
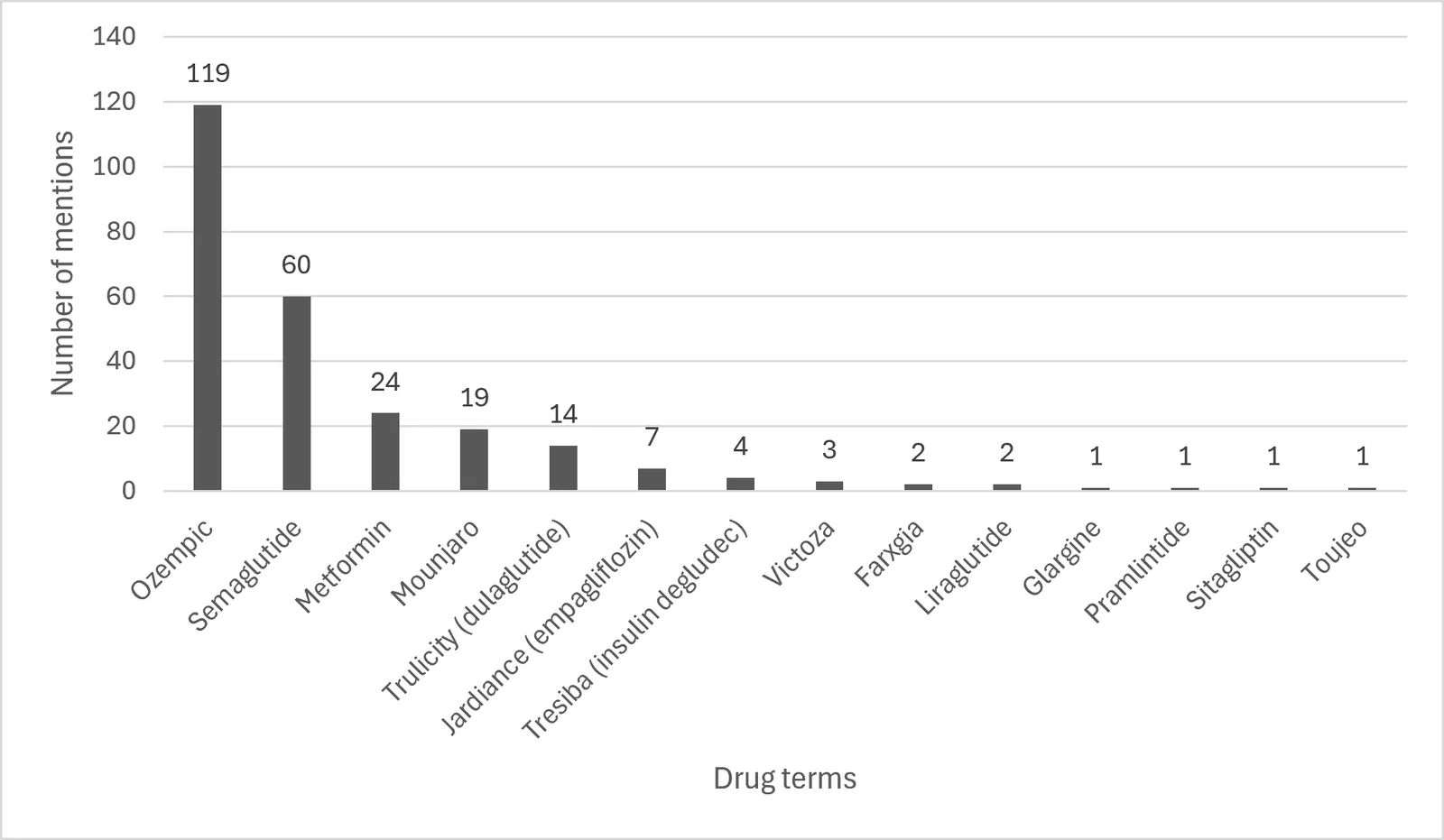
Top 14 drug terms mentioned in query 1b.

As shown in Figure 3, among the query 1b (including insulin and weight loss) online conversations, the most popular ADD mentioned was Ozempic (n=119). Semaglutides (n=60), the generic name for a GLP-1 receptor agonist, was the next most frequently mentioned drug online. Even though semaglutide and Ozempic share the same active ingredient, there are compounded versions of these drugs (compounded semaglutides) that are not FDA-approved and have been shown to cause medical complications for patients who use them, posing a concern for physicians [5,6,18]. Ozempic was used twice as often in this online discussion as the generic term semaglutide. The remaining drugs were mentioned less than half the number of times as Ozempic and semaglutides. Metformin (n=24), Mounjaro (n=19), and Trulicity (n=14) were mentioned as the next highest in this query. Ozempic was mentioned in 20% (119/609) of the posts in query 1b. When weight loss is included in the online ADD conversation, Ozempic and related semaglutides were the ADDs most frequently discussed online.

**Figure 4.**
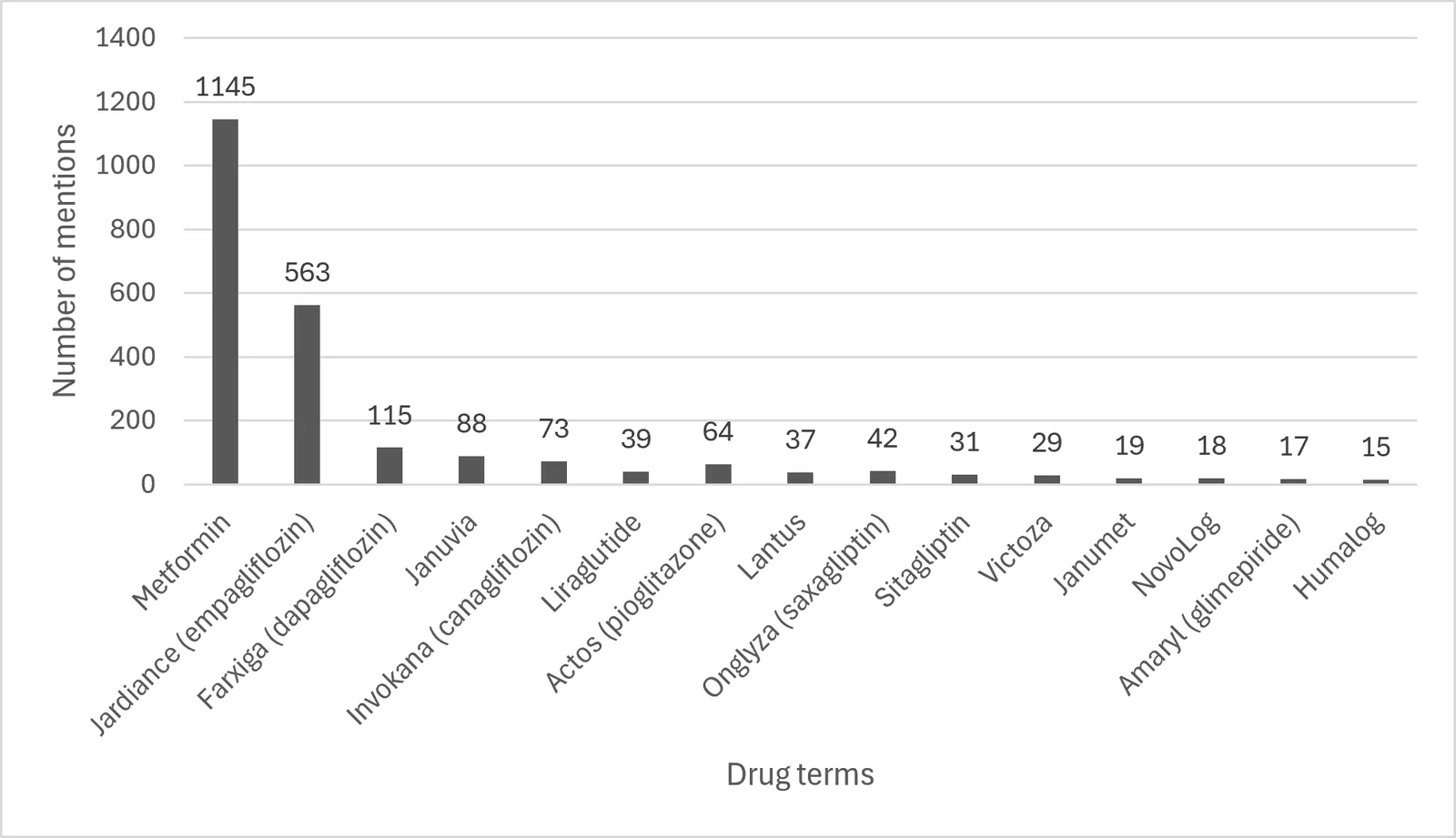
Top 15 drug terms mentioned in query 2a.

Figure 4 shows query 2a (excluding insulin and weight loss), metformin (n=1145) was still the most referenced antidiabetic medication. While not as heavily associated with weight loss as other antidiabetic medications, metformin has been shown to lessen weight gain in patients taking certain antipsychotic medications [19]. Jardiance (empagliflozin; n=563) was the next highly referenced antidiabetic medication used to treat type 2 diabetes. Farxiga (dapagliflozin; n=115) was the third highest referenced drug, followed by Januvia (n=88). Of these medications, Farxiga was the only medication approved to treat more than type 2 diabetes—it could also be used to prevent heart failure and chronic kidney disease [20]. Metformin was mentioned approximately 512.3 times per 1000 posts, meaning that almost half of the 2235 posts in query 2a mentioned metformin.

**Figure 5.**
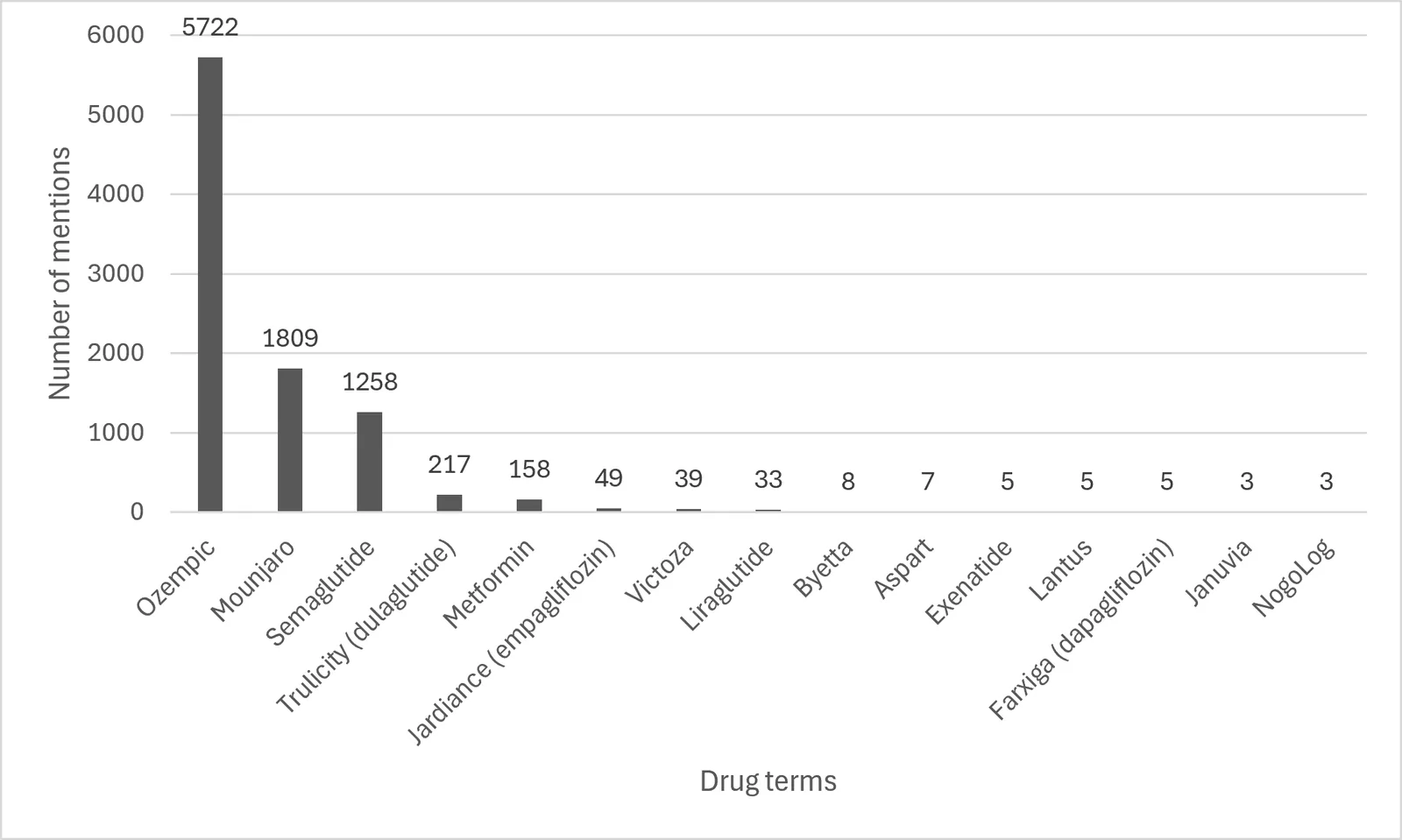
Top 15 drug terms mentioned in query 2b.

Within query 2b, Ozempic (n=5722) was the drug most mentioned, appearing in 28% (5722/20,258) of the posts as seen in Figure 5. The next most discussed ADD was Mounjaro (n=1809), appearing in 9% (n=1809) and semaglutides (n=1258) appearing in 6% (n=1258) of the posts in query 2b. Ozempic was mentioned approximately 282.5 times per 1000 posts, meaning that 30% of the posts in query 2b were discussing Ozempic. Brand names such as Ozempic, versus its generic name, semaglutide, are used interchangeably online; however, the brand name was used 4.5 times more online than the generic name. The greater recognition of Ozempic in online discussions suggests that patients and other individuals may be more familiar with Ozempic than with semaglutide.

When insulin was included in the online ADD conversation (query 1), metformin was the most popular drug referenced in query 1. In contrast, when weight loss was included in the conversation, regardless of insulin, Ozempic became the most frequently mentioned drug, surpassing metformin in the number of references. This finding underscores the noteworthy influence Ozempic has on the current ADD market.

### Physician Count

**Figure 6.**
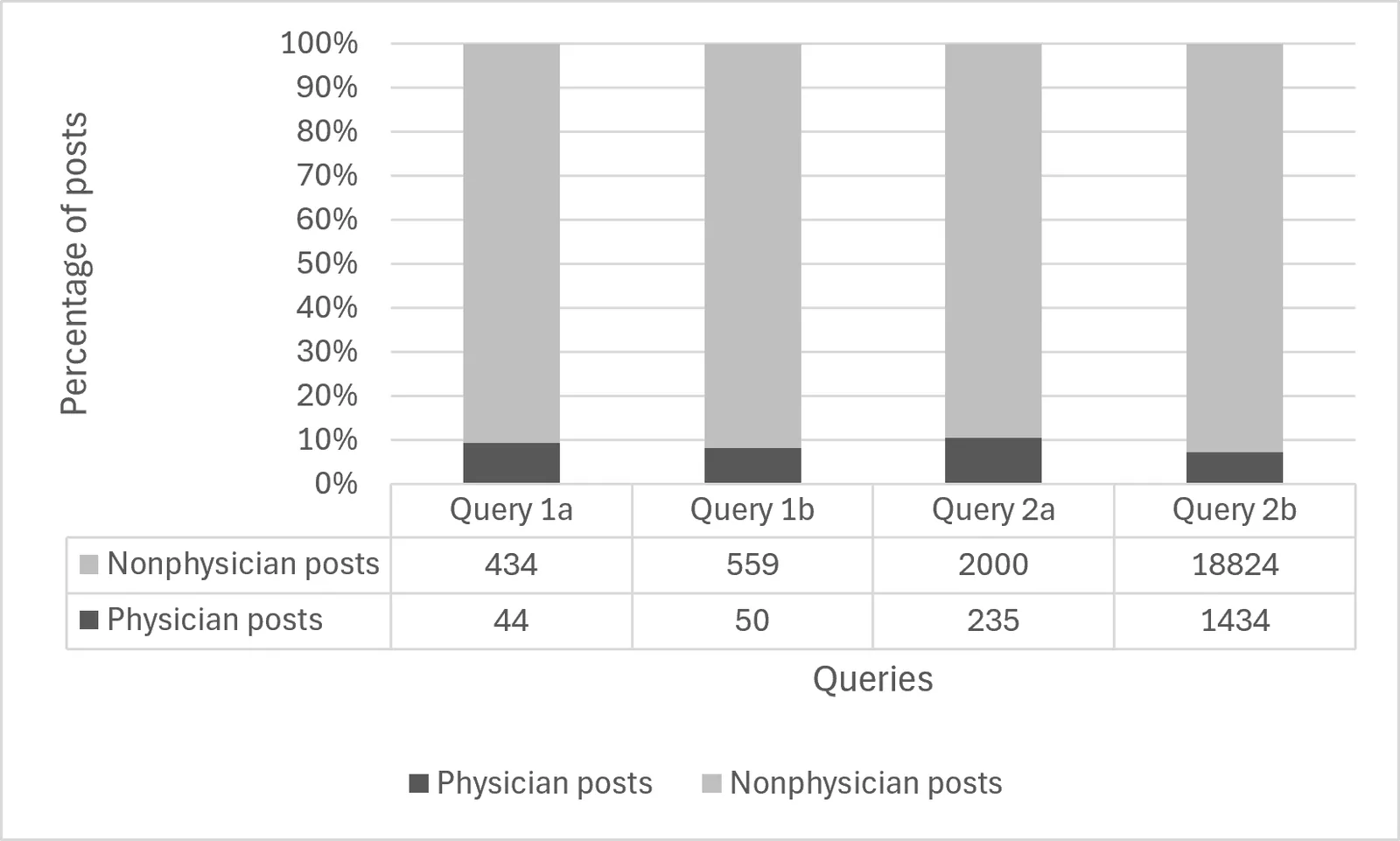
Percentage of posts by self-identified physicians.

Within query 1a (including insulin and excluding weight loss), 9.2% (44/478) of posts came from self-identified physicians. Within query 1b (including insulin and weight loss), 8.2% (50/609) of posts came from self-identified physicians. Query 2a (excluding insulin and weight loss) contained 10.5% (235/2235) of posts from self-identified physicians. Query 2b (excluding insulin and including weight loss) had 7.1% (1434/20,258) of the online conversation. Within each of the 4 query subsections, clinicians made up at most 10.5% of the conversation, meaning that most of the online conversation was not by medical professionals, as seen in Figure 6.

### Thematic Analysis

Query 1a focused on ADD-related posts that included insulin and excluded weight loss. Several key points emerged from this subset of the conversation. Using Synthesio’s Gen AI Trend Clusters capability, several prominent themes were identified, including insulin therapy, which is the gold standard for treating type 1 diabetes and more severe cases of type 2 diabetes. This involves regular insulin injections to help maintain proper body function. There was growing interest in online conversations about more innovative ways to administer insulin, such as patches or inhalation, which are less strenuous and taxing on the body. There was also interest in innovative insulin options that require only weekly injections rather than daily ones.

Individuals expressed concerns about access to affordable insulin treatments, including over-the-counter options that do not require a physician’s prescription. Consumers viewed over-the-counter options as more affordable and accessible than prescription alternatives. Additionally, there was a growing demand for preventative antidiabetic measures, such as improved access to healthy food, exercise, and better living habits, along with stronger patient advocacy to prevent hyperglycemia and type 2 diabetes. Many were concerned that the health care industry prioritized treating diabetes rather than focusing on preventing its onset.

In contrast, query 1b also included insulin but added weight loss to the discussion. Both query 1a and 1b highlighted significant conversations around innovative and less invasive antidiabetic treatments, particularly noninjectables such as metformin. Because weight loss was a focus, these discussions centered less on the cost of and access to medications and more on the desire for access to healthier lifestyles and physical well-being. Additionally, there was a strong interest in incorporating community support opportunities into antidiabetic treatment plans. Among the innovative treatment options, both patients and clinicians advocated for more digital health tools, enabling physicians to remotely monitor patients’ lifestyle behaviors and provide virtual care.

Query 2a excluded both insulin and weight loss posts from the online diabetes conversation, resulting in the emergence of vastly different themes. Notably, holistic medicine and personalized care were prominent topics, as many patients preferred less invasive, individualized treatments tailored to their specific diabetic symptoms. These treatments included vitamin and mineral supplementation, plant-based diets, integrated care (combining medicinal and holistic approaches), education, and community support groups. This trend contrasts sharply with conversations that include insulin and underscores the desire among many diabetic and prediabetic individuals for lifestyle-focused treatments aimed at preventing the symptoms associated with the disease. Finally, this query highlighted the impact of diabetes on mental health and the growing demand for treatments that incorporated psychotherapy.

Query 2b excluded insulin and included weight loss posts, which had the largest post count of all the queries. Within this query, patients highlighted the effectiveness of drugs such as Ozempic in treating their diabetic symptoms as well as the weight loss benefits this drug is associated with. Similarly, patients and health care providers agreed that strict diet and exercise routines increase the efficacy of these medications. Additionally, the demand for Ozempic and similar medications is rapidly increasing as they can now be used to primarily treat weight loss, not only diabetes. The demand for these drugs has brought vocalized disdain from diabetic patients about the strain this puts on companies to keep these medications in stock for diabetic patients. There is also the fear that the increase in popularity of weight loss–associated drugs will increase the price of the medication and decrease insurance coverage. Diabetic patients worry that prior authorization will pose issues in receiving the drugs in the future, and physicians and health care professionals lament unaffordable prices for drugs such as Ozempic and Mounjaro in the United States in comparison to other countries.

### Physician Themes

Clinician input was manually analyzed by focusing on posts from users who self-identified as physicians through terms such as MD, doctor, physician, or clinician in their name or bio. This small subset of posts highlighted several important conversations and concerns physicians had regarding antidiabetic medications.

In query 1a, posts from self-identified physicians made up 9.2% (44/478) of the results. This group praised the use of insulin treatments requiring only weekly injections instead of daily ones and supported the increased use and availability of noninsulin medications for treating type 2 diabetes. Additionally, clinicians emphasized the importance of healthy lifestyles, including proper nutrition and exercise, as lifestyle choices significantly affected obesity and type 2 diabetes symptoms. Query 1b, making up 8.2% (50/559) of the posts, revealed that physicians associated weight loss with the mitigation of type 2 diabetes symptoms, and that Ozempic could aid in this process. Additionally, clinicians believe health care resources should prioritize the prevention of type 2 diabetes through lifestyle behaviors rather than its management after onset.

In contrast, within query 2a, which comprised 10.5% (235/2000) of the posts, physicians highlighted both the positive and negative aspects of metformin. While metformin offers benefits beyond insulin regulation for type 2 diabetes patients, such as stabilizing white blood cell counts, reducing inflammation, preventing cancer, prolonging lifespan, and supporting proper immune responses, physicians also cautioned that discontinuing metformin in geriatric patients with diabetes may accelerate cognitive decline.

Query 2b mirrors query 1b, with physicians making up 7.1% (1434/18,824) of the conversation. As in query 1b, physicians consistently emphasized the importance of combining dietary and active lifestyle behaviors with innovative medications. Uniquely, query 2b also highlighted how Ozempic and similar GLP-1 RA drugs have revolutionized the antidiabetic treatment experience. When paired with proper nutrition and exercise, these medications help patients lose weight and effectively manage diabetic symptoms.

## Discussion

### Principal Findings

Our findings reveal that within the diabetic medication landscape, there is a notable focus on insulin alternatives such as Ozempic, reflecting their current medical popularity. This surveillance study revealed that conversations excluding insulin made up 95% of the diabetes conversation online, meaning insulin accounted for a small proportion of current public discourse on diabetes. Topics within this insulin-excluded conversation predominantly revolve around weight loss–associated drugs, which offer additional benefits beyond insulin regulation, enhancing their appeal. This surveillance study also revealed a current focus in the health care market: laypersons—both patients and nonpatients—dominate the conversation on ADDs, whereas physicians contribute only a small portion. FDA-approved GLP-1 RA medications such as Ozempic and Mounjaro are prescribed by physicians to treat weight loss [9]. This current pharmaceutical landscape is reflected online, and this study monitors and highlights this current public interest in ADDs for treating weight loss in patients without diabetes [21]. Wellness advocates, diabetes advocacy groups, and health-related accounts also significantly contribute, often emphasizing the potential risks and considerations for those using GLP-1 RAs such as Ozempic. Unlike insulin-based treatments, these drugs offer additional benefits beyond managing insulin levels, contributing to their rising popularity. Furthermore, when weight loss is included in the conversation online, Ozempic and semaglutides are mentioned far more frequently than any other ADD brand name. When weight loss is part of the discourse, mentions of Ozempic and semaglutides surpass those of other ADDs. This suggests a significant public interest in ADDs for nondiabetic use. The findings underscore the evolving role of weight loss associations in ADD discussions, reflecting the growing use of these medications for weight management in nondiabetic patients, both as Wegovy and through off-label prescriptions. This strongly indicates that online discussions about ADDs, excluding insulin, often focus on drugs associated with weight loss. This study elucidates the current popularity of ADDs in online conversation as a microcosm for our medical and health care landscape.

Although X (formerly Twitter) is a single platform and is not comprehensive of the entire US population, its robust usership within the United States makes the platform a powerful tool for observing public discourse. It is imperative that further studies be conducted across various prominent social media platforms to expand the knowledge on this emerging public health issue. Because this study did not use demographic data, it did not qualify as human subjects research; therefore, demographic data are not available to represent characteristics of the average user. As an observational study, trend analyses were not used; rather, the study focused on actual counts to measure the prevalence of the online discussion. This study used X data available in 2023, and future studies will continue to survey the progression of the ADD landscape.

### Limitations

It is important to note that although this study provides valuable insights that add to the previous literature, limitations still exist. First, the queries used did not isolate the term “semaglutide” from “compounded semaglutide.” A manual analysis of the posts found that semaglutide and compound semaglutides were used interchangeably by patients and laypersons online. Therefore, the counts of semaglutides mentions in 2023 include both “compounded semaglutide” and “semaglutide” references.

Additionally, some clinicians may have been excluded from the study because they used “doctor” in their social media bio or username, which was not included as a keyword in this study to avoid including individuals with PhD degrees in the conversation results as noise. Therefore, medical doctors who self-identified on social media only as “doctor” without “md” or “medical” also in their bio would not have been captured in the physician sample set.

Furthermore, the data were constrained to English-language posts; therefore, this study could only survey and report on patients and individuals who speak and write English, excluding non–English-speaking medical populations.

Finally, these surveillance results show the emerging landscape of these medications and their rise in popularity. The underlying mechanisms may include, but are not limited to, increased pharmacological options, direct-to-consumer marketing, efficacy, and affordability. This surveillance study provides a foundation for future research to better understand these mechanisms.

### Conclusions

This study highlighted the current popularity of ADDs, specifically GLP-1 RAs, in the online diabetic medication conversation. GLP-1 RA medications dominate conversation (~23,000 posts), and these medications were the most referenced for treating diabetes. Online laypersons expressed apprehension about accessing these medications for purposes other than diabetes treatment, causing concern among diabetic patients, who voiced these concerns online. Many diabetics are worried that the exponential increase in demand for ADDs for nondiabetic users for cosmetic purposes would increase the burden on supply and cause shortages or price hikes for those who need these medications to maintain body homeostasis [22]. Ozempic was originally FDA-approved to treat type 2 diabetes but has been prescribed off-label and was recently approved under the name Wegovy to promote weight loss in patients with obesity [8,18]. These federal approvals mark a key moment in the health care industry, reflecting a popularity in ADDs use. Additionally, laypersons used the term “semaglutide” and “compounded semaglutide” interchangeably online, often using the term “semaglutide” to refer to the non–FDA-approved compounded forms of these medications used to treat weight loss, which could cause some confusion about the actual drug being referred to, the former being FDA approved and the latter not FDA approved. Although the terms were used interchangeably, Ozempic was used overwhelmingly more often in online diabetic conversations than the generic term semaglutide. This evidence offers an insight into the fact that individuals and patients were more aware of Ozempic and its uses than of semaglutides. Physicians primarily used the term “compounded semaglutide” to voice their concern over patients going to medical spas to receive non–FDA-approved medication to try and mimic the weight loss effects and putting their health at risk to achieve weight loss. The FDA has published work supporting this online concern by noting that there have been anecdotal physician reports of patients experiencing gastroparesis as a result of using compounded semaglutides [6]. Physicians noted that antidiabetic medications are profoundly serious drugs that require changes in lifestyle and behavior to have the most positive effects during and after treatment.

## Supplementary material

10.2196/87590 Multimedia Appendix 1 Query structure.

10.2196/87590 Multimedia Appendix 2 Query terms.

10.2196/87590 Multimedia Appendix 3 Noninsulin diabetic medications and misspellings.

10.2196/87590 Multimedia Appendix 4 Self-identified physician post isolation.

## Abbreviations

ADD: antidiabetic drug
FDA: Food and Drug Administration
GLP-1 RA: glucagon-like peptide 1 receptor agonist
