# Supplementary material for: Social Media Discussions of Anti-Diabetic Drugs and the Popularity of GLP-1 Therapies: Content Analysis
Source: JMIR Diabetes. 2026 Aug 26;11:e87590. doi: 10.2196/87590 (PMC13515349; doi:10.2196/87590)
Supplement: Supplementary file 1 [file diabetes-v11-e87590-s002.docx]

## Appendix 1: Query Terms

### Query 1a: + Insulin (-) Weight loss

((Diabetes OR Anti-diabetes OR Diabet OR "Diabetic med" OR "Anti-diabetic" OR antidiabet OR "Diabetes drug" OR "Diabetes med" OR Endocrinology OR Endocrin OR Endocrinologist)

AND

(Insilin OR Insulan OR Insulin OR Insuling OR Insulins OR Insullin OR Insuln)

NOT

(weight OR ("weight loss") OR (weightloss) OR pandemic OR covid19 OR covid OR corona* OR coronavirus OR Sema* OR Semaglutide OR Dula OR Dulaglutide OR Dulga* OR Terz* OR terzaputide OR Trulic* OR Trulicity OR Monj* OR Mounj* OR Mounjaro OR Ozem* OR Ozempic OR Rybelsus OR Rybel* OR Rybelsis))

### Query 1b: + Insulin + Weight loss

(((Diabetes OR Anti-diabetes OR Diabet OR "Diabetes med" OR "Diabetic med" OR "Anti-diabetic" OR antidiabet OR "Diabetes drug" OR "Diabetes med" OR Endocrinology OR Endocrin OR Endocrinologist)

AND

(Insilin OR Insulan OR Insulin OR Insuling OR Insulins OR Insullin OR Insuln))

NEAR (weight OR weightloss OR Sema* OR Semaglutide OR Dula OR Dulaglutide OR Dulga* OR Terz* OR terzaputide OR Trulic* OR Trulicity OR Monj* OR Mounj* OR Mounjaro OR Ozem* OR Ozempic OR Rybelsus OR Rybel* OR Rybelsis))

NOT ("pandemic" OR "covid19" OR "covid" OR "corona*" OR "coronavirus"))

### Query 2a: (-) Insulin (-) Weight loss

((Diabetes OR Anti-diabetes OR Diabet OR "diabetes med" OR "Diabetic med" OR "Anti-diabetic" OR antidiabet OR "Diabetes drug" OR "Diabetes med" OR Endocrinology OR Endocrin OR Endocrinologist)

NOT

(Insilin OR Insulan OR Insulin OR Insuling OR Insulins OR Insullin OR Insuln OR weight OR ("weight loss") OR (weightloss) OR pandemic OR covid19 OR covid OR corona* OR coronavirus OR Sema* OR Semaglutide OR Dula OR Dulaglutide OR Dulga* OR Terz* OR terzaputide OR Trulic* OR Trulicity OR Monj* OR Mounj* OR Mounjaro OR Ozem* OR Ozempic OR Rybelsus OR Rybel* OR Rybelsis)

### Query 2b: (-) Insulin + Weight loss

((Diabetes OR "Anti-diabetes" OR Diabet OR "Diabetic med" OR "Diabetes med" OR "Diabetic med" OR "Anti-diabetic" OR antidiabet OR "Diabetes drug" OR "Diabetes med" OR Endocrinology OR Endocrin OR Endocrinologist)

AND

(weight OR weightloss OR "weight loss" OR Sema* OR Semaglutide OR Dula OR Dulaglutide OR Dulga* OR Terz* OR terzaputide OR Trulic* OR Trulicity OR Monj* OR Mounj* OR Mounjaro OR Ozem* OR Ozempic OR Rybelsus OR Rybel* OR Rybelsis))

NOT

(Insilin OR Insulan OR Insulin OR Insuling OR Insulins OR Insullin OR Insuln OR pandemic OR covid19 OR covid OR corona* OR coronavirus OR fruit OR Lizzo OR sugar OR "artificial sweetener" OR sweetener OR soda OR cavity OR "sugary drink" OR Trump OR Anderson OR "cb wellness" OR gestational OR maternal OR pregnant OR handler, OR Osbourne OR Richards OR Kardashian OR celebrity OR fasting OR "physical activity" OR keto* OR sleep OR celeb* OR pet OR dog OR feline OR vet OR veti* OR carb* OR acne OR caffeine OR coffee OR vax OR vacc* OR vegan OR vegetarian)
