# Supplementary material for: Social Media Discussions of Anti-Diabetic Drugs and the Popularity of GLP-1 Therapies: Content Analysis
Source: JMIR Diabetes. 2026 Aug 26;11:e87590. doi: 10.2196/87590 (PMC13515349; doi:10.2196/87590)
Supplement: Supplementary file 2 [file diabetes-v11-e87590-s003.docx]

## Appendix 2: Non-insulin Diabetic Medications & Misspellings

(Actos OR Albiglutide OR Alogliptin OR Amaryl OR Apidra OR Aspart OR Avandia OR Basaglar OR Bydureon OR Byetta OR Canagliflozin OR Chlorpropamide OR Dapagliflozin OR Degludec OR Determir OR Diacetate OR Dulaglutide OR Empagliflozin OR Ertugliflozin OR Ertugliflozin OR Exenatide OR Farxiga OR Fiasp OR FlexPen OR Formamet OR Glargine OR Gliclazide OR Glimepiride OR Glipizide OR Glucophage OR Glucotrol OR Glulisine OR Glumetza OR GlynaseGylburide OR Humalog OR Humolog OR Humulin OR Humulog OR Invokana OR Isophane OR Janumet OR Januvia OR Jardiance OR Kanazo OR Lantas OR Lantis OR Lantus OR Levemir OR Linagliptin OR Liraglutide OR Lispro OR Metaform* OR Metaglip OR Metform* OR Micronase OR Micron* OR Miglitol OR Nateglinide OR Nesina OR Novalog OR Novol* OR Onglyza OR Orinase OR Oseni OR Pioglitazone OR Pramlintide OR Prandin OR Repaglinide OR Repaglinide OR Riomet OR Rosiglitazone OR Ryzodeg OR Ryzodeg OR Saxagliptin OR Sitagliptin OR Starlix OR Steglatro OR Tol-Tab OR Tolazamide OR Tolbutamide OR Tolinase OR Toujeo OR Tradename OR Tradjenta OR Trajenta OR Trandate OR Tresiba OR Victoza OR Vildagliptin OR Zonulin)
