# Supplementary material for: Social Media Discussions of Anti-Diabetic Drugs and the Popularity of GLP-1 Therapies: Content Analysis
Source: JMIR Diabetes. 2026 Aug 26;11:e87590. doi: 10.2196/87590 (PMC13515349; doi:10.2196/87590)
Supplement: Supplementary file 3 [file diabetes-v11-e87590-s004.docx]

## Appendix 3: Self-Identified Physician Tweet Isolation

“MD” OR “md” OR “m.d.” OR “M.D.” OR “medical doctor” OR “med doc”
